# Supplementary material for: 3D atomic structure from a single X-ray free electron laser pulse
Source: Nat Commun. 2024 Feb 1;15:970. doi: 10.1038/s41467-024-45229-8 (PMC10834439; doi:10.1038/s41467-024-45229-8)
Supplement: Supplementary file 1 — Supplementary Information [file 41467_2024_45229_MOESM1_ESM.pdf]

Supplementary Information

for

3D atomic structure from a single X-ray free-electron laser pulse

by

G. Bortel, M. Tegze, M. Sikorski, R. Bean, J. Bielecki, C. Kim, J. Koliyadu, F. Koua, M. Ramilli, A. Round, T. Sato, D. Zabelskii, G. Faigel

In the first part of this document we describe the complete evaluation process, from the measured raw image to the 3D real space structure. Parts of this procedure were already described in our previous publications [1,2,3]. This part is followed by a section about the effect of radiation damage and another about the prospective applications.

Supplementary Note 1: Evaluation process from raw Kossel line patterns to electron densities

Calibration to photon counts. The raw detector data represents the total charge measured in each pixel of the 4M pixel Jungfrau detector [4]. This is proportional to both the photon energy and the number of photons detected. Based on the nominal photon energy (fluorescent energy of the excited element of the sample), these pixel values were converted to calibrated photon counts. The calibrated detector images for GaAs and GaP single-shot structure determination are shown in Supplementary Figure 1. They consist of 8 modules of 8 chips each, with some gaps between them. The calibrated images contain 20/120 and 200/800 fluorescent photons/pixel/pulse at the edges/center of the 4M Jungfrau detector placed at 120 mm from the sample for GaAs and GaP, respectively.

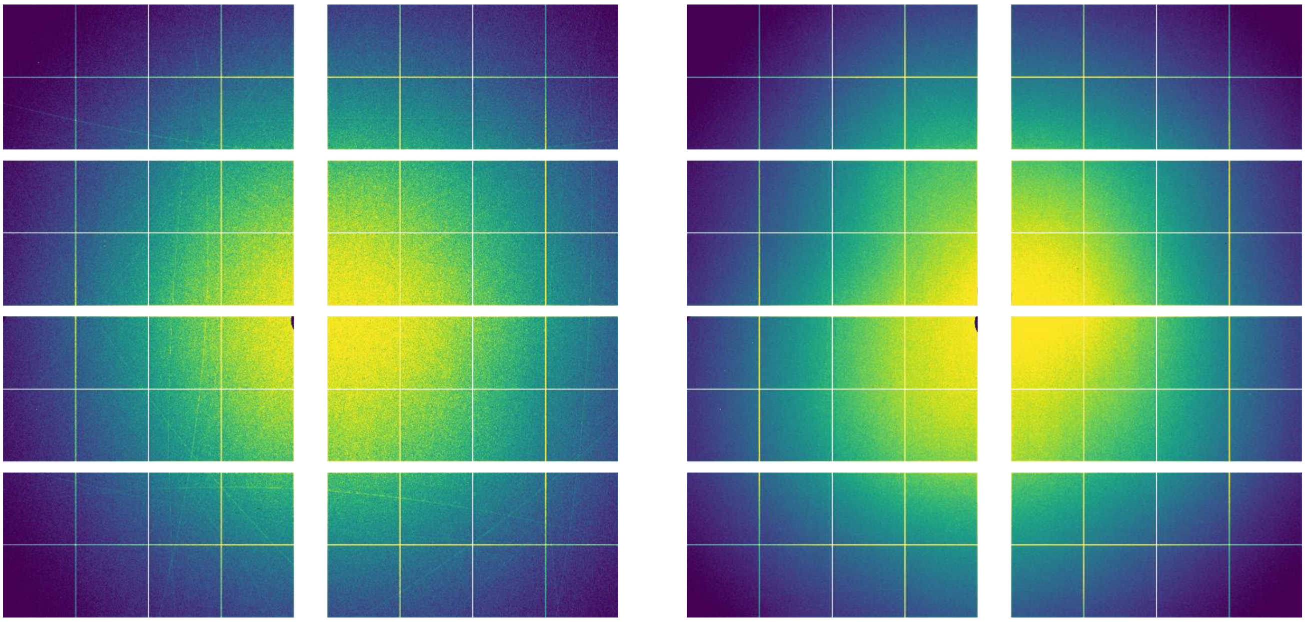

Supplementary Figure 1. Calibrated detector images for GaAs and GaP. Source data are provided.

Background removal and normalization. A continuous decrease in intensity from the center to the edge of the detector is caused by three factors: scattering of the incident beam on the gas in the experimental chamber, geometrical effects caused by the flat detector and absorption of the fluorescent beam in the sample and in the gas present in the chamber. This smooth, slowly changing background intensity is a disturbing factor when analyzing the sharp Kossel lines and was removed. The background determined by median filtering was used to normalize the calibrated image. This puts the patterns on a general scale, where 1 corresponds to the level of unmodified fluorescent intensity, and the surplus and deficit

intensity in the Kossel line profiles is related to this level. Supplementary Figure 2 shows these background-corrected and normalized images. The Kossel lines are clearly visible, even to the naked eye.

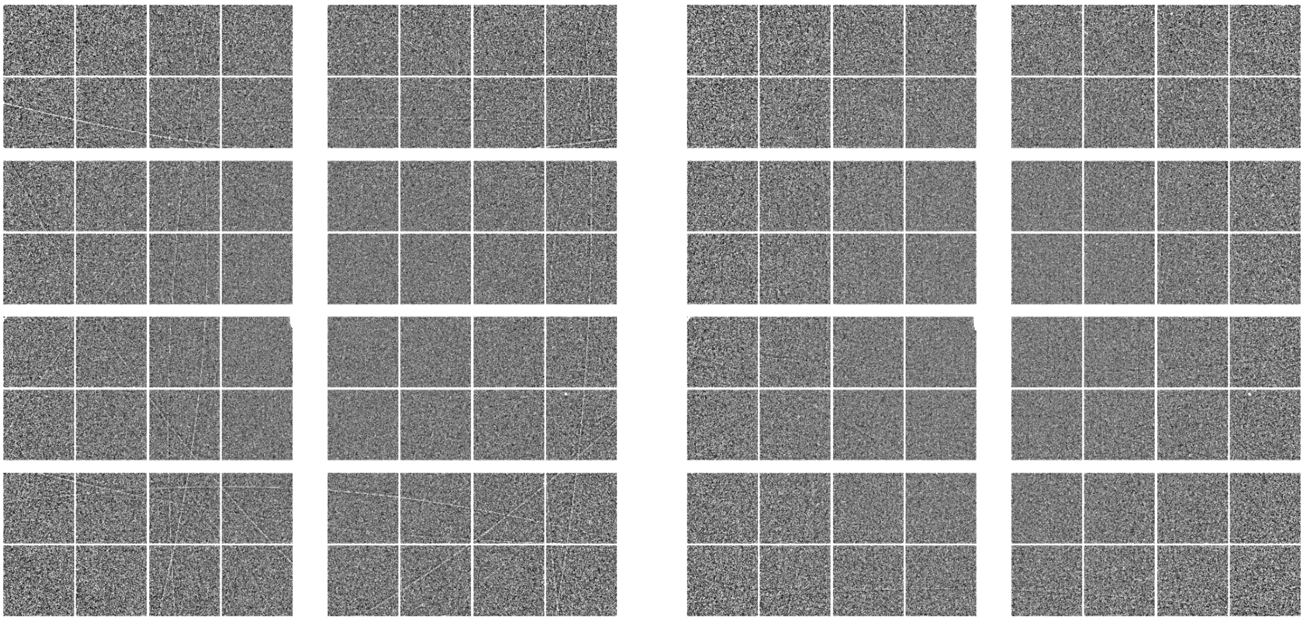

Supplementary Figure 2. Background corrected and normalized images for GaAs (left) and GaP (right). Source data are provided.

Kossel line indexing. Our procedure to find both the unknown lattice and orientation of the crystal, *i.e.* autoindexing the pattern was already described earlier [3]. Briefly, conic sections are individually fitted to the lines on the observed image yielding the axis and opening angle of the individual cones. By assuming a common apex for all cones, this information is converted to scattering vectors for each Kossel line, defining a reciprocal lattice point in the reciprocal space. The set of these reciprocal lattice points is indexed with some well-established indexing procedure used in single crystal diffraction yielding the orientation, lattice and reciprocal lattice vector indices.

Geometry refinement. A special software tool was developed that allows simultaneous refinement of all relevant parameters: detector module positions, crystal lattice parameters and crystal orientation matrix. The result of this process in a magnified region of the pattern is shown in Supplementary Figure 3., where color-overlaid lines show the calculated centerline of the $K\alpha_{1,2}$ and $K\beta$ Kossel lines, matching the lines on the image with pixel precision. This refinement also indicates that the lattice is cubic, since a single lattice constant and 90° lattice angles perfectly describe all Kossel lines.

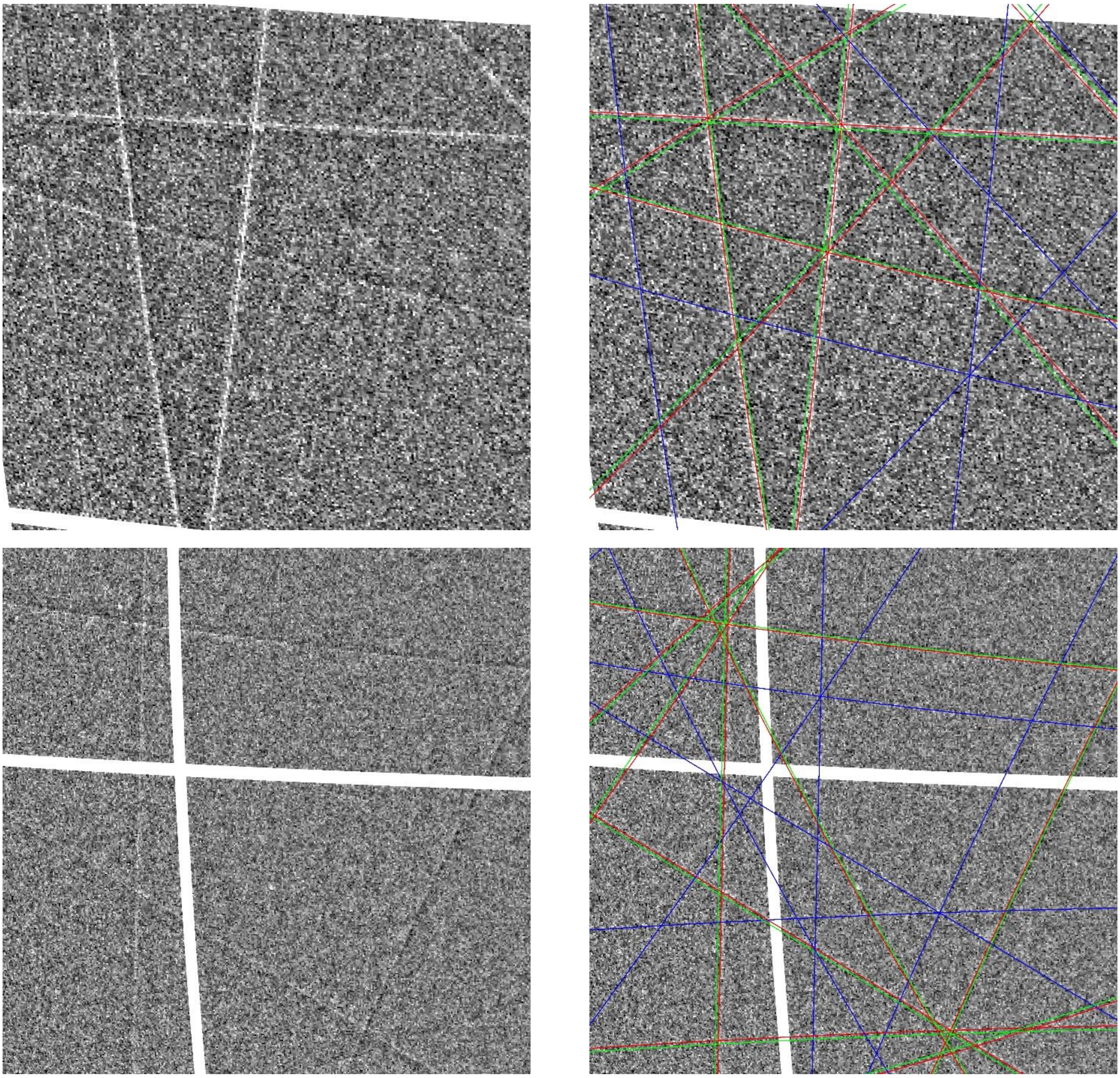

Supplementary Figure 3. A magnified region of the patterns without and with overlaid indexed lines for all 3 emission energies (red, green and blue) up to 1.4 Å resolution for GaAs and GaP.

Profile extraction. The next step in the evaluation process is the extraction of the line profiles. In principle, the line profile is changing along a line *i.e.* around a Kossel cone, since the angle of the involved waves with the sample surface is changing. However, this change is small on the relatively small solid angle covered by the detector. Therefore, we integrated the images along a Kossel line for different cone opening angles to obtain an average line profile. These profiles as a function of the opening angle can be brought to a common scale, the emission wavelength, using Bragg's law. These extracted line profiles together with the number of contributing pixels up to 1.4 Å resolution are shown in Supplementary Figure 4. The deviation from the average fluorescence level of the strongest Kossel lines was (+20%, –8%) and (+4%, –3%) for GaAs and GaP, respectively. It is worth comparing these amplitudes with estimated Poisson statistics of the extracted profiles: The average ~80 and ~500 photons/pixel raw calibrated intensity (Supplementary Figure 1) for GaAs and GaP respectively, and the typical 300 contributing detector pixels to a single Kossel line profile point (Supplementary Figure 4 bottom panels) gives 24000 and 150000 photons/profile point estimated values. Their relative

statistical uncertainty, 0.0065 and 0.0026 is well below the line amplitudes, indicating statistically meaningful results.

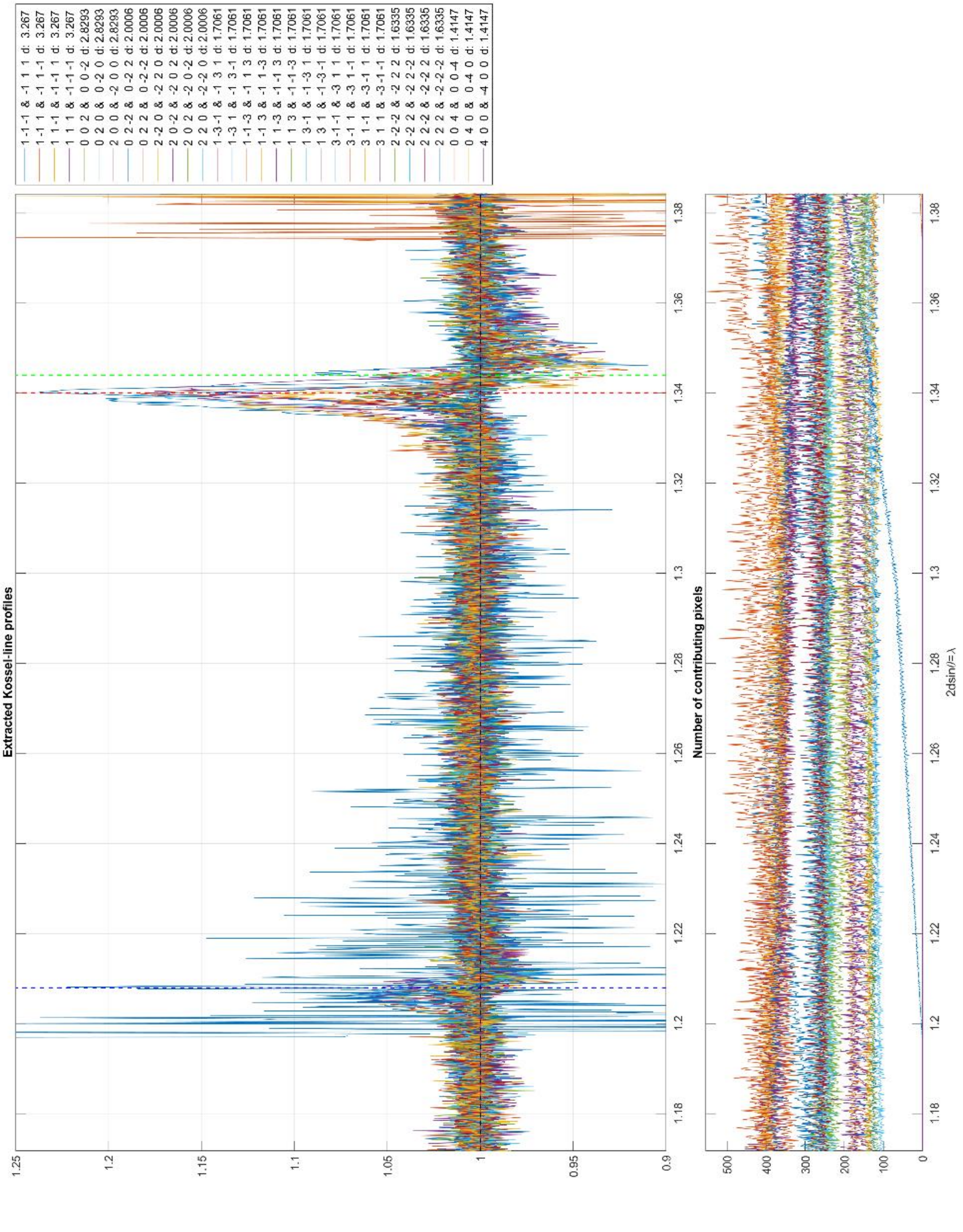

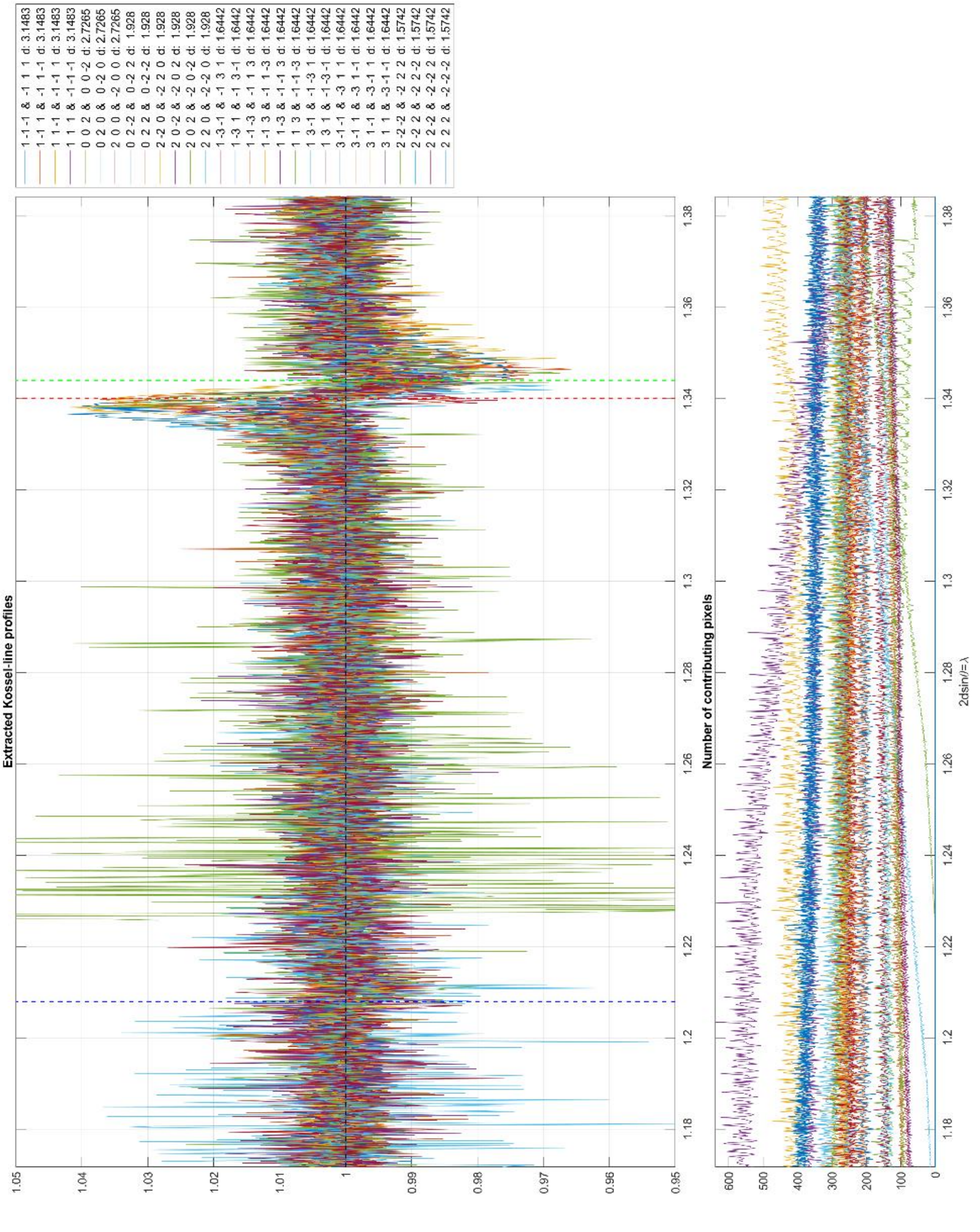

Supplementary Figure 4. Extracted Kossel line profiles up to 1.4 Å resolution for GaAs (on the preceding page) and GaP (on this page). It can also be seen on the bottom panels, how the statistical noise increases where the integration range is not completely covered by the detector image and the number of contributing pixels decreases to zero. Source data are provided.

The inventory of the reflections in the step of profile extraction is the following: The ~0.7Å resolution limit defined by the emission line wavelength and the ~5.55Å average lattice parameter of the structures allow ~2000 cubic reflections within the resolution sphere $[(4\pi/3\,(2\pi/0.7)^3)/(2\pi/5.55)^3]$. In the profile extraction, the Friedel pairs, the opposite halves of a Kossel cone were not distinguished, they contributed to a single profile, reducing the number of reflections to ~1000. The complete absence of Kossel lines in the extracted profiles for all mixed parity indices indicates a volumetric systematic absence, that proves the face centering of the conventional cubic lattice. These reflections were taken as zero, further decreasing the number of usable reflections to ~250. The profiles of all these Kossel lines were attempted to be extracted from the image, but some of the Kossel cones are not covered by the solid angle of the detector at all, some have just a fraction on the detector and only a few high index cones fall completely on the image. The number of contributing pixels to the profile integration (shown also in Supplementary Figure 4) is the strongest factor that affects the existence, statistics and quality of the extracted profile. A critical selection of these left us with ~100 usable Kossel line profiles for further analysis.

Profile fitting. The line profile is used to obtain the phases and the amplitudes of the structure factors. In Supplementary Figure 5 typical low, medium and high index profiles are shown for illustration (it is the same as Figure 4 and 5 of the paper).

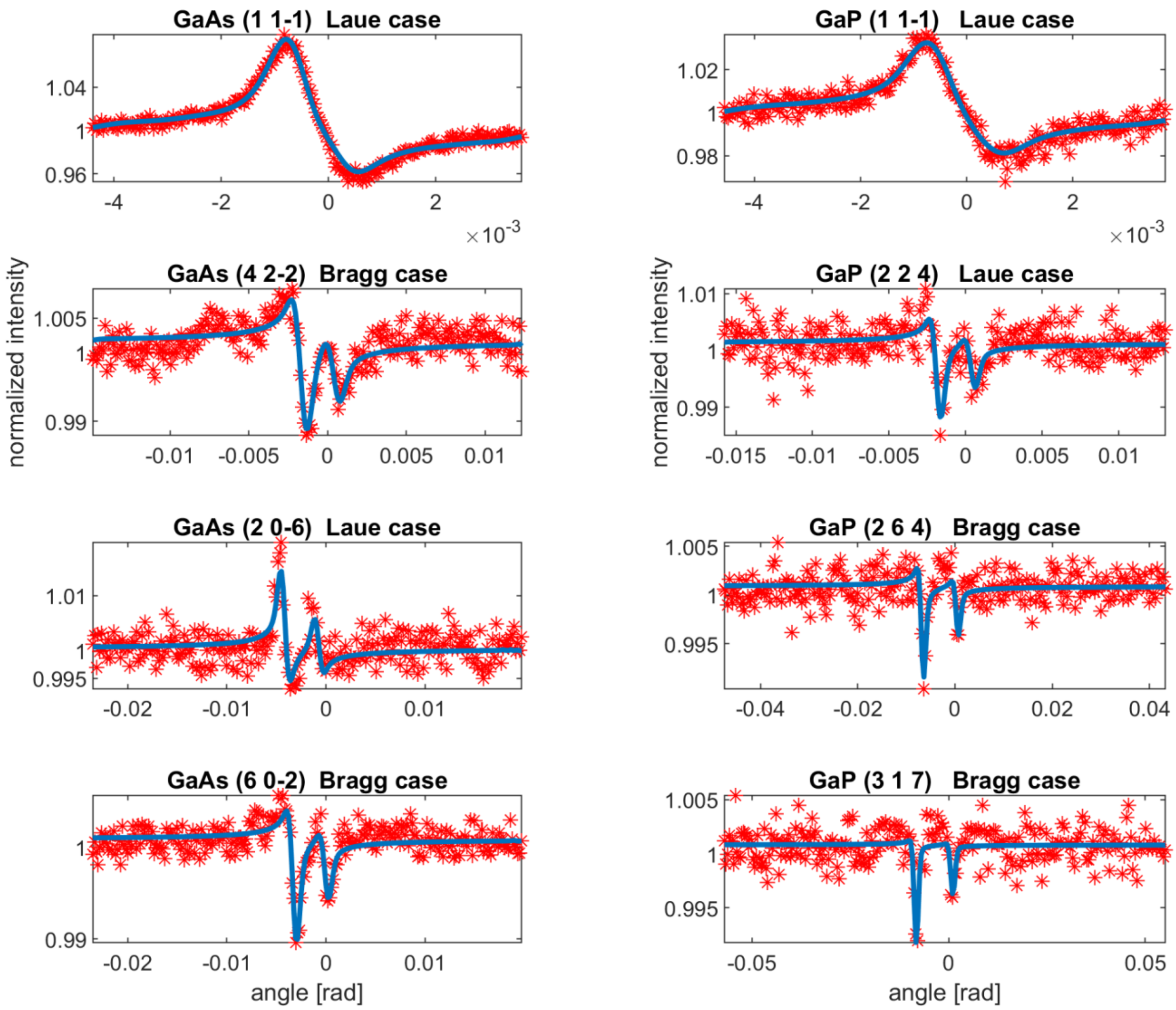

Supplementary Figure 5. Theoretical fit to the Kossel line profiles for some selected reflections for GaAs and GaP, respectively. Red stars indicate the profile data points extracted from the experimental Kossel line pattern. Blue lines indicate the fit of the theoretical model to the experimental profile. Reproduced from the main text to illustrate the complete evaluation process in this document. Source data are provided.

For the description of the line profiles, we followed the work of Hannon and Trammell [5,6,7]. In their theoretical treatment, they used the real space approach, which starts from the Darwin description of dynamical diffraction of x-rays in single crystals. However, they extended the theory for the case of inside sources. The theoretical treatment is quite involved, and we do not repeat it here. We give only the final formulas used in the evaluation. Our notation mostly follows the convention of [5,6,7] for easier understanding. The solution to this diffraction problem splits into two parts, depending on the geometry of the experiment: (i) Laue case and (ii) Bragg case. In the terminology of dynamical diffraction, these cases are explained with the help of the crystal surface, the crystallographic layers and the beam directions. The two cases are illustrated in Supplementary Figure 6.

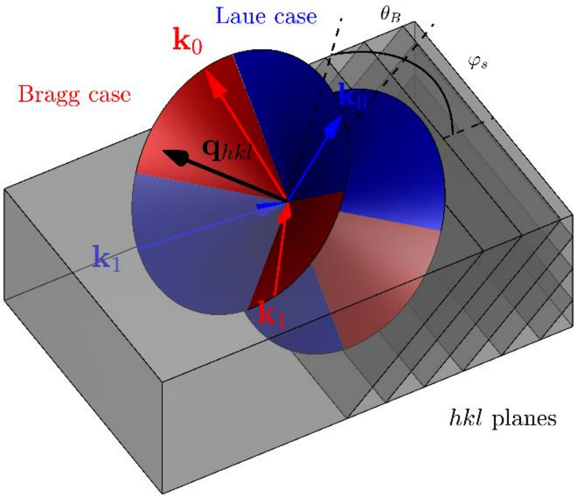

Supplementary Figure 6. Illustration of Bragg and Laue cases. In the Bragg case, the wave vectors coupled by the reflection are on one side of the crystal surface, while in the Laue case are on opposite sides. To see how the Bragg and Laue cases change with the surface angle, see the Supplementary Movie 2. The figure and movie are adapted from our earlier publication [Faigel, G., Bortel, G. & Tegze, M. Experimental phase determination of the structure factor from Kossel line profile. *Scientific Reports* **6**, 22904 (2016).].

The variation of the intensity as a function of the deviation from the Bragg angle is given by (1) and (2) for the Laue and Bragg cases, respectively. These formulas express the contribution of a source atom located at $\mathbf{r}_i$ in the M_j-th layer to the reflection *hkl*, at a given wavelength λ.

For the Laue case:

$$I(\delta) = B\left|\left(1+\frac{\nu}{\beta}-C\frac{F^{01}}{\beta}\right)e^{iM_j(\alpha+\beta)}+\left(1-\frac{\nu}{\beta}+C\frac{F^{01}}{\beta}\right)e^{iM_j(\alpha-\beta)}\right|^2, \qquad (1)$$

where

$\alpha = \frac{1}{2}(F^{00}+F^{11})$,
$\beta = (\nu^2+F^{01}F^{10})^{1/2}$,
$\nu = \delta+\frac{1}{2}(F^{00}-F^{11})$,
$C = e^{i(\mathbf{k}_0-\mathbf{k}_1)\mathbf{r}_i}e^{-i(\alpha+\delta)}$.

For the Bragg case:

$$I(\delta) = B\left|\frac{1+im^*CF^{01}}{Q}e^{iM_j(\alpha+\beta+gd-\delta)}\right|^2, \qquad (2)$$

where

$\alpha = \frac{1}{2}(F^{00}-F^{11})$,
$\beta = (\nu^2-F^{01}F^{10})^{1/2}$,
$\nu = \delta+\frac{1}{2}(F^{00}+F^{11})$,

$$m^* = \frac{i\left(1-e^{2i(M-M_j+1)\beta}\right)}{\nu+\beta-(\nu-\beta)e^{2i(M-M_j+1)\beta}},$$

$Q = \frac{\nu+\beta}{2\beta}\left(1 - \frac{\nu-\beta}{\nu+\beta} e^{2i(M-M_j+1)\beta} + i\big(M - M_j + 1\big)(\nu - \beta)\big(1 - e^{2iM_j\beta}\big)\right),$

$C = e^{i(\mathbf{k}_0-\mathbf{k}_1)\mathbf{r}_i}.$

$\delta = |\mathbf{k}_0| d \frac{\sin(2\theta_\mathrm{B})}{2\sin(\varphi_1)}(\theta - \theta_\mathrm{B})$ is related to the angular deviation from the kinematical Bragg angle θ_B; M is the total number of layers, M_j is the serial number of the layer containing the source atom below the surface of the crystal toward the detector, $\mathbf{k}_0$ is the direct wave wave-vector, $\mathbf{k}_1$ denotes the wave-vector of the wave which is scattered into $\mathbf{k}_0$ satisfying the Bragg condition ($\mathbf{k}_0 = \mathbf{k}_1 + \mathbf{q}_\mathrm{hkl}$ where $\mathbf{q}_\mathrm{hkl}$ is the reciprocal lattice vector of the reflection under consideration and $|\mathbf{k}_0| = |\mathbf{k}_1| = 2\pi/\lambda$), g is the projection of $\mathbf{k}_0$ to the normal of the crystal surface, $\mathbf{r}_j$ denotes the position of the source atom relative to the origin of the unit cell, B is a normalization factor, which describes the intensity of the Kossel line relative to the total off-Bragg intensity and can be calculated from the fluorescent yields. θ is the rocking angle, φ_1 (φ_0) is the angle between $\mathbf{k}_1$ ($\mathbf{k}_0$) and the crystal surface, d stands for the interplanar spacing, F^{00}, F^{11}, F^{01} and F^{10} are the planar scattering amplitudes (for definition see equations (7) and (8) in [6]).

The total intensity variation is obtained by summing the contributions of all layers containing source atoms. Since we intend to determine the phase (Φ) and magnitude (A_0) of the structure factor we recall the dependence of F^{01} on Φ *and* A_0 [6]: $F^{01} = A_0/\sin(\varphi_1)e^{i\Phi}$. Note, that in traditional crystallography, the choice of the origin of the unit cell affects the phase of reflections via a phase-factor. However, in our measurement the source atom fixes the origin and through this, the phases of the Kossel lines. Profiles were fitted to the lines according to equations (1) and (2). In principle, there are four parameters to fit: F^{01}, F^{10}, F^{00} and F^{11}. Examining equations (1) and (2) we see that F^{00} and F^{11} forward scattering amplitudes determine the shift of the diffraction lines relative to the kinematical Bragg angle. Their absolute value is proportional to the number of electrons in the unit cell, and their imaginary part is determined by the absorption. These values are known and given by the sample composition. In general, their values are small; the shifts are in the arcsec range. Since we cannot measure absolute angular positions with this precision by our setup, we fit the position of the peaks for every line independently. However, the fitted values of the peak positions are not used in the structure solution. For the structure factor determination, the important parameters are F^{01} and F^{10}. Their phase determines the shape of the Kossel lines. By definition, F^{01} and F^{10} are not independent; $F^{01} = A_0/\sin(\varphi_1)\times e^{i\Phi}$ while $F^{10} = A_0/\sin(\varphi_0)\times e^{-i\Phi}$. This means that we have to fit the Kossel line profile by a minimum of 3 parameters: A_0, Φ and a position parameter. However, in practice we have one more fitting parameter determined by the experimental conditions: the line broadening. It comes from three factors: the energy width of the fluorescent lines, the imperfection of the crystal, and the angular resolution of the experimental setup. The energy width of the fluorescent lines can be precisely given but the other two factors change from line to line depending on the geometry and, in practice, they cannot be easily derived. Estimating their contributions, we found that the crystal imperfection and the angular resolution of the setup dominate, and the smallest contribution comes from the energy width of the fluorescent lines. We took into account the line broadening by convoluting the theoretical lines with a Gaussian. An average value for the φ_1 and φ_0 angles of the wavevectors with the sample surface (more precisely, the ratio of their sines) were taken from the indexing and geometry refinement step for the fraction of the Kossel line falling on the detector.

Therefore, all together the line profiles were fitted by four parameters: line position, line broadening, amplitude of structure factor and phase of the structure factor. Since the statistics of the measurements were relatively poor because of the single pulse used for the collection of a full pattern, the fitting procedure had to be done very carefully. A brute force approach *i.e.* starting from a random set of all parameters and fitting all in one iteration process does not converge. Therefore, we used a 3-stage process: first, we found approximate starting parameters for the iteration. This was done by choosing

estimated values for the position, broadening and magnitude of the lines, meanwhile mapping the phase (Φ) from 0–360° in 20° steps. We selected the starting phase at the value where the line shape was closest to the measured one. This was done for all lines by visual inspection. In the second stage, we fixed the phase and performed a 3-parameter nonlinear minimization of the sum of squared differences of the measured and calculated profiles. This gave the experimental parameters: the width of the Gaussian, and the positions of the lines, and a starting value for the amplitudes to the last iteration. In the third stage, we fixed the experimental parameters and iterated for the phase and amplitude.

The result of the above fitting procedure for the two experimental parameters is the following: the broadening is between 30-80 times, while the line shift is -10 to +20 times the theoretical line width. These are in accordance with the expectations based on the detector geometry. As we mentioned earlier, we did not use these parameters in the structure solution. The two important parameters concerning the structure solution are the phase and magnitude of the structure factor. Most of the phases of the structure factors were within 20 degrees of the theoretical values. However, some phases had as large as 40 degrees errors. We show in Supplementary Figure 7 the measured phases against the theoretical values. The measured amplitudes of the structure factors are shown in Supplementary Figure 8 together with the theoretical values.

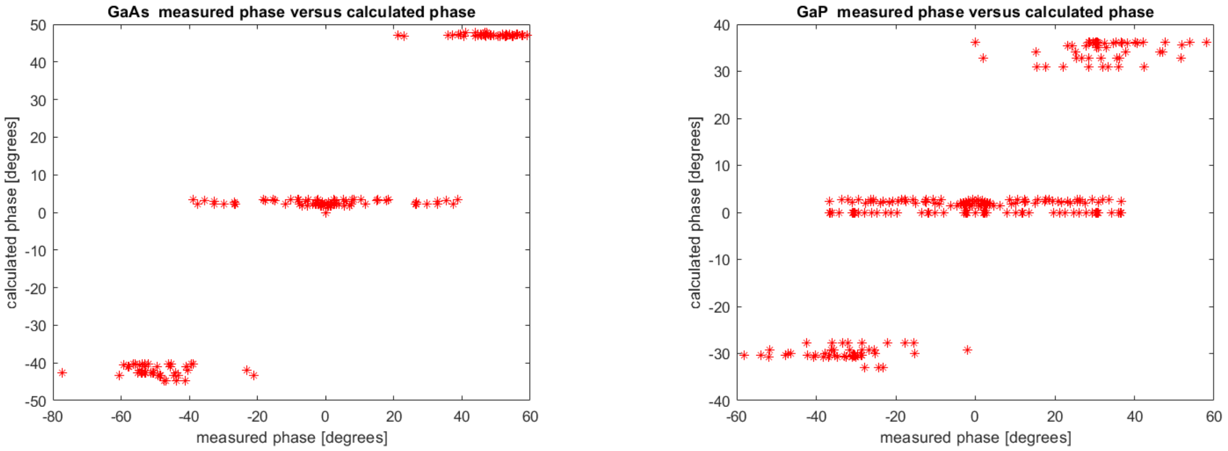

Supplementary Figure 7. Correlation Figures of the measured and calculated structure factor phase angles for GaAs and GaP. Source data are provided.

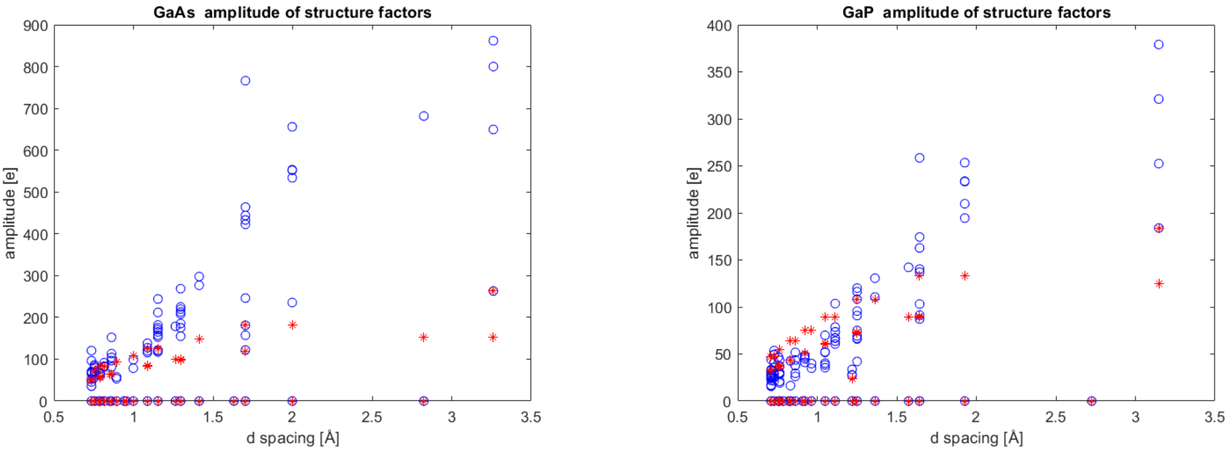

Supplementary Figure 8. Measured (blue circles) and calculated (red stars) structure factor amplitudes for GaAs and GaP. Source data are provided.

Fourier-synthesis. The last step of evaluation is the inversion of the structure factors to real space electron density, the atomic structure. We did this by putting the complex structure factors to the measured reciprocal lattice points, extended the reciprocal lattice with the Friedel pairs (equivalent of supposing real electron density) and for the forward direction, which we could not measure we put the total charge in the unit cell given by the composition of the sample. For smoothness, we extended the Fourier transformation range by three times the size of the range of the measured indices. The unknown lattice points were taken as zeros. This 3D complex matrix was Fourier transformed, resulting in a real electron density distribution, shown in Supplementary Figure 9.

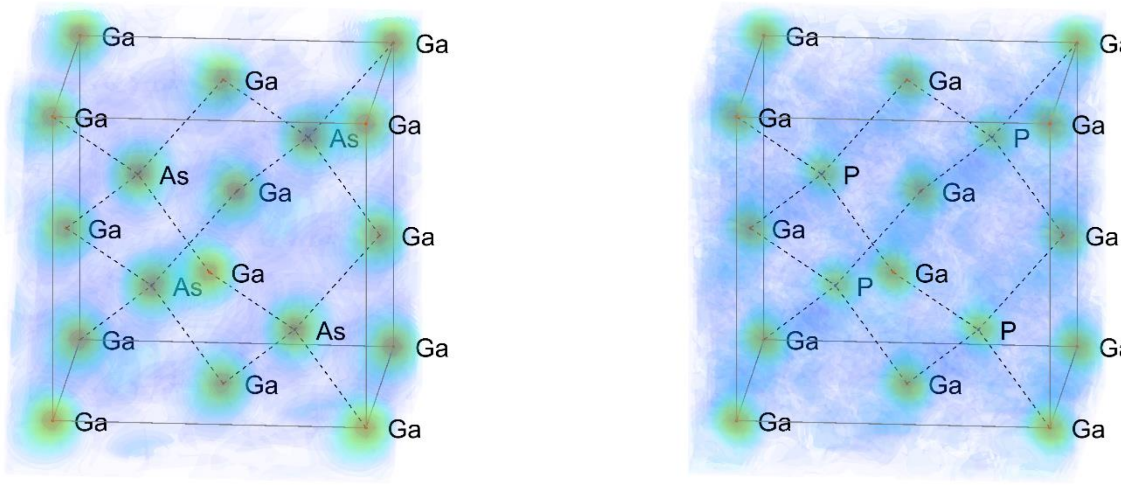

Supplementary Figure 9. Electron density distribution obtained by direct Fourier synthesis for GaAs and GaP. For 3D information see the Supplementary Movies 3 and 4. Source data are provided.

In order to estimate the error in atomic positions and electron density (peak heights and width where atoms are found), we made model calculations on the same grid as the measured data were given using the same number and *hkl* index of structure factors that were measured. We compared this to the density reconstructed from the measured data. In Supplementary Figure 10 we show the reconstructed electron density of GaP for the theoretical structure factors and for the measured structure factors using isosurface plot. On this type of plot, it is easier to recognize the differences between the two reconstructions. It is clear that the “sizes” of the atoms are larger in the reconstruction from the measurement. This is caused by the error in the measured structure factors. The difference in width is about 15%. However, the positions of the peaks do not change within the grid size, which is determined by the FFT range. Further, we also checked the integrated intensity within the peaks, and we found that the deviation compared to the theoretical values was in the range of 10%. We made the same calculations for GaAs and found similar results. This explains why we could not distinguish the Ga and As atoms in our measurements; the error in the integrated intensity was larger than the difference in the atomic numbers.

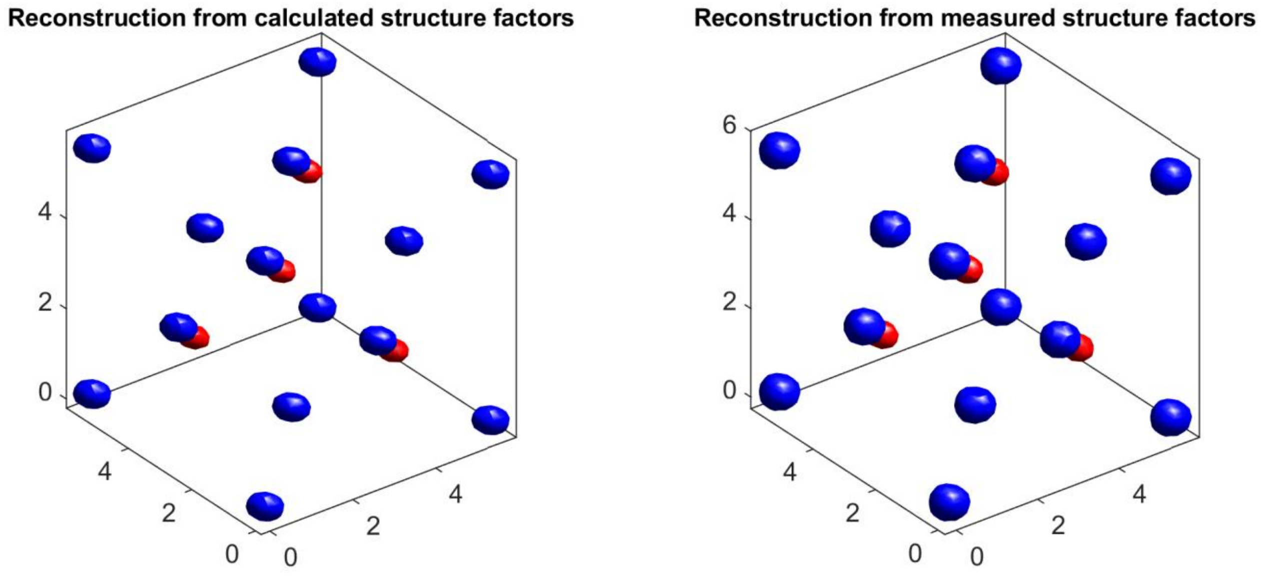

Supplementary Figure 10. Comparison of real part of reconstructed electron densities with calculated and measured structure factors using the same grid and set of reflections. In both figures the isosurface is drawn at 50% level of peak density. Blue surfaces are around the Ga atoms, red surfaces are around the P atoms. Source data are provided.

Supplementary Note 2: Discussion of radiation damage

In XFEL experiments, the extremely high number of photons interacting with the sample in a small area and in a very short time causes radiation damage. This, in most of the cases is fatal, resulting in evaporation of the sample (Supplementary Figure 11). However, there is a possibility to overcome this problem by measuring the structure before the atoms have time to move. This was suggested first in 2000 [8]. Later, many theoretical works investigated radiation damage (see for example [9]), and the idea was also experimentally proved. In our case, the most relevant experiments are the serial femtosecond crystallography (SFX) experiments, where small crystallites are introduced into the beam. Using SFX, several structures were determined at atomic resolution. There were studies aiming to find the effect of pulse length and pulse intensity on the structure solution using SFX. In an early work [10] it was found that in the 1 μm spot size range, at about 10 fs pulse length and at $\sim 10^{12}$ photons/pulse region the effect of radiation damage resulted in higher background, but the diffraction spots could be measured, and the structure could be determined without problem. In our experiment the spot size was 25 μm, which is about 10 times the spot size used in SFX measurements. The other beam parameters are the same as in an average SFX experiment. Therefore, the deposited energy on unit area is 100 times smaller than in a typical SFX. Since our measurement is also x-ray diffraction (although using inside sources), we do not expect deformation in the diffracted intensity pattern. Further, even the increase of the background is negligible because of the 100 times less deposited energy in unit area. Besides the radiation damage nonlinear effects could also appear, caused by the high energy density in the sample. This type of effects was studied in [11]. In this work the authors calculate the effect of high energy density on the imaginary part of the atomic scattering factor f”. They find significant changes for samples used in SFX experiments. However, we can neglect this effect because we have about 100 times smaller energy density on our sample. Although, after the pulse we see a hole where the beam hits the sample (Supplementary Figure 11), the formation of this hole happens in a much longer time scale than the measurement of the elastically scattered fluorescent radiation. However, the radiation damage prevents the measurement of the original structure on the same spot of the sample using a second pulse.

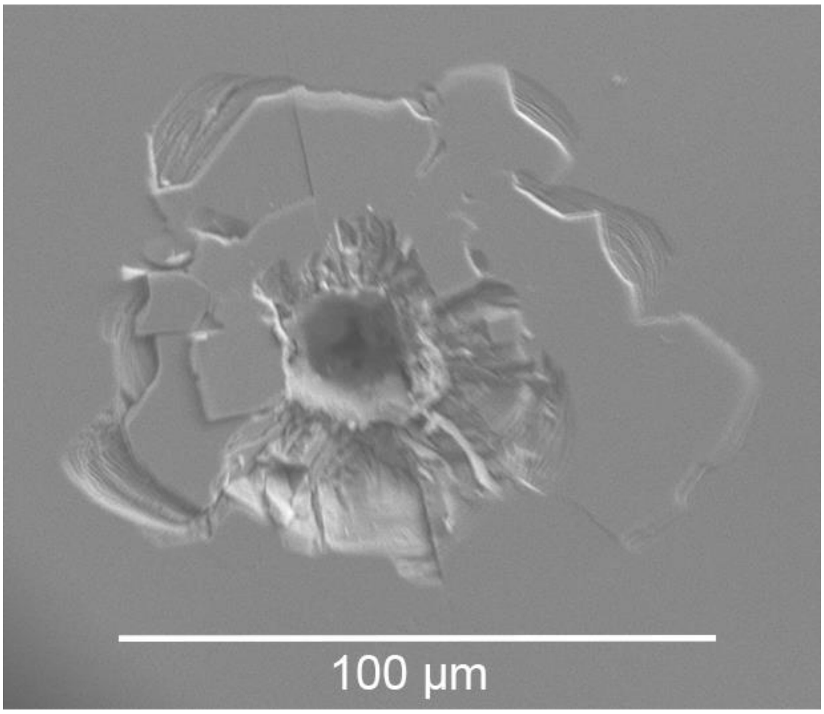

Supplementary Figure 11. Scanning electron microscope image of the GaAs sample after hit by a single XFEL pulse. The hole in the center reflects the ~25µm diameter of the beam. Around the hole we see the deterioration of the crystal surface caused by the heat shock.

Supplementary Note 3: Prospects for possible applications

In this section we summarize the conditions necessary for measuring Kossel line patterns at XFEL-s. These conditions give guidelines for those who intend to use our method for structural studies. Based on the conditions in the last part of this section we give few specific examples for application. First, we discuss the sample parameters and then the beam parameters.

The sample has to contain at least one heavy element in the unit cell. Heavy element means emission energy larger than 5 keV. Smaller energy will result in lower spatial resolution. The sample size has to be larger than 10 µm. At smaller sample size one has to use smaller focal spot (see beam parameters later in the text), which may result in higher background and nonlinear effects modifying the atomic scattering factors leading to distortion in the diffracted intensity distribution. This would increase the error in the phase determination from the Kossel line profile. In our demonstration experiment, we used samples with simple structures, only a few atoms in the unit cell. In the next part, we will discuss prospects for more complicated structures.

First, take a sample containing one heavy atom and many other atoms, which are not excited. In this case, the structure solution is going the same way as we did in the presented two cases. However, as the number of atoms increases in the unit cell one has to measure more Kossel lines. As a rule of thumb, one should measure about 10 Kossel lines for each atom in the unit cell.

Our next example is when we have a sample containing two different heavy atoms, both of which are excited. Although, in principle, it is possible to experimentally separate the emission lines if their energies are far enough, however, we would not rely on this. Instead, we should sort out the Kossel lines corresponding to the two emission energies by their different positions in the measured 2D image. This was already demonstrated in our earlier experiment performed at synchrotron on GaAs [1], where both the Ga and As emission lines were used. If the lines are sorted out this way, the solution of the

structure is even more stable, because it works as if two independent measurements with different energies were done.

The third example is a sample in which there are two inequivalent sites of the same atom type in the unit cell and many other atoms, which are not excited. In this case, the phase of the structure factors cannot be determined without additional knowledge. We have to know the relative position of the two excited atoms. In principle, this could be determined by fitting the relative position together with the phases for all lines in one iteration process. However, this would make the solution very complicated. Therefore, we do not recommend to measure this types of samples before working out the proper procedures for the above mentioned iteration process.

One more remark about the sample composition: so far, we mostly discussed what type of fluorescent atoms the sample has. However, the measured pattern contains information on the non-fluorescent atoms too. The question arises: what we can tell about those. Similarly, to traditional single crystal diffraction measurements, the lighter elements have smaller weights. Therefore, we can measure on samples containing light elements (for example biological samples), but we do not expect to see lighter elements than carbon. Further, as we mentioned in connection to the first example, we have to measure more Kossel lines for more complicated structures, which makes the evaluation more difficult. The reason is that Kossel lines are not point like (as Bragg peaks) but lines on the detector surface. Therefore, it will be more difficult to separate them if there are too many. This results in more stringent technical limits on the detector and evaluation software than we have in traditional crystallography. A further point, which one has to take into account when measuring biological samples, is the crystal quality, which is usually not too good. Mosaic crystals would wash out the line shape resulting in the loss of phase information. However, this does not limit structure solution more than the missing phase in traditional diffraction. In this case, one can use the well-established single crystal algorithms to solve the structure.

Turning to the beam parameters, it has to be tailored to the given experiment. First, the beam energy should be chosen to excite the heavy atom we want to use for producing the Kossel lines. It is advantageous if we excite only a single atom type. The beam size should be about the same as the sample size. It is important to excite the largest number of atoms possible. The pulse should contain as many photons as we can get. Going below 10^{12} photons/pulse will severely limit the complexity of the structure we can determine. The length of the pulse should be in the 10-50 fs range to avoid deterioration of the Kossel lines because of radiation damage.

Below we will indicate what type of studies would mostly benefit from single pulse structure determination. We have already mentioned in the main text those general areas, the extremely non-ambient condition studies, which would benefit most. Here we give two specific examples.

(a) Many studies aim to find the atomic structure of matter at very high pressures (for example pressures present at the interior of planets like Earth, Jupiter etc.), which is difficult to statically maintain. In these cases, we cannot do pump-probe experiments in easily repeatable and well-defined way. However, we can make this large pressure for a very short time (ms-ns). There is no method which could determine the atomic structure at these cases from one single measurement. Our approach could do it.

(b) Other example is very high magnetic fields (above 30 Tesla). If one intends to study matter in very high magnetic fields, which cannot be produced as a static field, but can be produced as one very short pulse, again there is no method which could determine the 3D atomic arrangement induced by this field. These types of investigations include superconductivity, strongly correlated and quantum critical systems, frustrated quantum magnets etc.

Last, we would like to mention one more possible application area, time resolved pump-probe experiments at XFEL. Presently these types of experiments are done in SFX mode. In this case one has to measure at every time point tens of thousands of samples. This takes a long beam time. If the samples, which we intend to study satisfy the conditions for Kossel measurement, we have to take only single shots at every time point (or a few shots taking into account the stochastic nature of the XFEL pulses and the less than 100% hit rate). This would shorten measuring times significantly. Therefore, it would increase the output of these very expensive facilities.

Supplementary References

1. Faigel, G., Bortel, G. & Tegze, M. Experimental phase determination of the structure factor from Kossel line profile. *Scientific Reports* **6**, 22904 (2016).
2. Bortel, G., Faigel, G., Tegze, M. & Chumakov, A. Measurement of synchrotron radiation excited Kossel pattern. *J. Synch. Rad.* **23**, 214–218 (2016).
3. Bortel, G. Tegze, M. & Faigel, G. Constrained geometrical analysis of complete K-line patterns for calibrationless auto-indexing. *J. Appl. Cryst.* **54**, 123–131 (2021).
4. JUNGFRAU (adJUstiNg Gain detector FoR the Aramis User station) detector https://www.psi.ch/en/lxn/jungfrau.
5. Hannon, J. P. & Trammell, G. T. Mössbauer diffraction. II. Dynamical theory of Mössbauer optics. *Phys. Rev.* **186**, 306–325 (1969).
6. Hannon, J. P., Carron, N. J. & Trammell, G. T. Mössbauer diffraction. III. Emission of Mössbauer γ rays from crystals. A. General theory. *Phys. Rev. B* **9**, 2791–2809 (1974).
7. Hannon, J. P., Carron, N. J. & Trammell, G. T. Mössbauer diffraction. III. Emission of Mössbauer γ rays from crystals. B. Dynamical solutions. *Phys. Rev. B* **9**, 2810–2831 (1974).
8. Neutze, R., Wouts, R., van der Spoel, D., Weckert, E., Hajdu, J. Potential for biomolecular imaging with femtosecond x-ray pulses. *Nature* **406**, 752–757 (2000).
9. Jurek, Z., Faigel, G. & Tegze, M. Dynamics in a cluster under the influence of intense femtosecond hard X-ray pulses. *European Physical Journal D* **29**, 217–229 (2004).
10. Barty A. et al. Self-terminating diffraction gates femtosecond X-ray nanocrystallography measurements. *Nature Photonics* **6**, 35–40 (2012).
11. Son, S. K., Chapman, H. N. & Santra R. Multiwavelength anomalous diffraction at high X-ray intensity. *Physical Review Letters* **107**, 218102 (2011).
