# Supplementary material for: 3D atomic structure from a single X-ray free electron laser pulse
Source: Nat Commun. 2024 Feb 1;15:970. doi: 10.1038/s41467-024-45229-8 (PMC10834439; doi:10.1038/s41467-024-45229-8)
Supplement: Supplementary file 3 — Description of Additional Supplementary Files [file 41467_2024_45229_MOESM3_ESM.pdf]

Description of Additional Supplementary Information

File name: Supplementary Movie 1

Description: Supplementary Movie for Figure 1. Formation of Kossel lines.

File name: Supplementary Movie 2

Description: Supplementary Movie for Supplementary Figure 6. Animation of Bragg and Laue cases

File name: Supplementary Movie 3

Description: Supplementary Movie for Figure 6 and Supplementary Figure 9. 3D rendering of reconstructed electron density for GaAs.

File name: Supplementary Movie 4

Description: Supplementary Movie for Figure 6 and Supplementary Figure 9. 3D rendering of reconstructed electron density for GaP
